# New directions in mapping the Earth’s surface with citizen science and generative AI

**DOI:** 10.1016/j.isci.2025.111919

**Published:** 2025-01-30

**Authors:** Linda See, Qingqing Chen, Andrew Crooks, Juan Carlos Laso Bayas, Dilek Fraisl, Steffen Fritz, Ivelina Georgieva, Gerid Hager, Martin Hofer, Myroslava Lesiv, Žiga Malek, Milutin Milenković, Inian Moorthy, Fernando Orduña-Cabrera, Katya Pérez-Guzmán, Dmitry Schepaschenko, Maria Shchepashchenko, Jan Steinhauser, Ian McCallum

**Affiliations:** 1 Novel Data Ecosystems for Sustainability (NODES) Research Group, International Institute for Applied Systems Analysis (IIASA), Laxenburg, Lower Austria 2361, Austria; 2 Department of Geography, University at Buffalo, The State University of New York, Buffalo, NY 14261, USA; 3 Department of Landscape Architecture, Biotechnical Faculty, University of Ljubljana, Jamnikarjeva 101, 1000 Ljubljana, Slovenia

**Keywords:** Earth sciences, Environmental science, Remote sensing, Cartography

## Abstract

As more satellite imagery has become openly available, efforts in mapping the Earth’s surface have accelerated. Yet the accuracy of these maps is still limited by the lack of *in situ* data needed to train machine learning algorithms. Citizen science has proven to be a valuable approach for collecting *in situ* data through applications like Geo-Wiki and Picture Pile, but better approaches for optimizing volunteer time are still required. Although machine learning is being used in some citizen science projects, advances in generative artificial intelligence (AI) are yet to be fully exploited. This paper discusses how generative AI could be harnessed for land cover/land use mapping by enhancing citizen science approaches with multi-modal large language models (MLLMs), including improvements to the spatial awareness of AI.

## Graphical abstract

**Figure:**
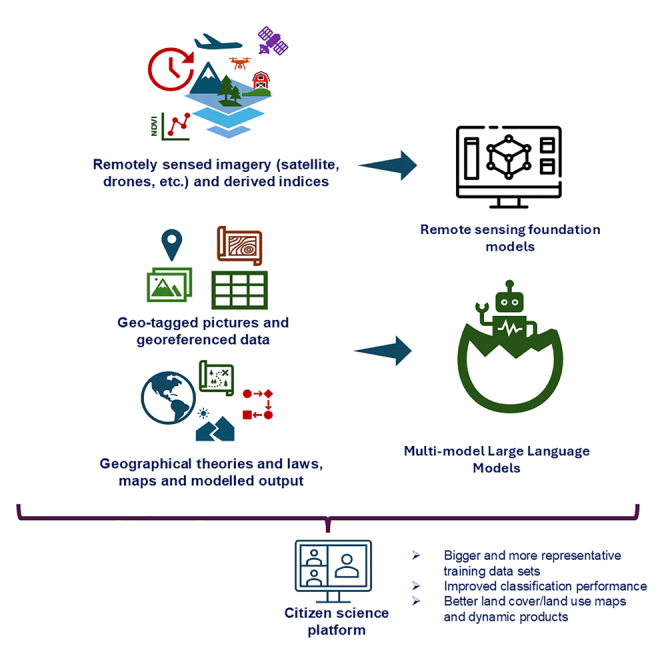

## Introduction

Monitoring of the Earth’s surface is crucial for quantifying the Earth’s carbon balance, climate mitigation potentials, and the risk of overshooting tipping points.^1^ Achievement of the United Nations (UN) Sustainable Development Goals (SDGs) and other targets set out in multi-lateral agreements also rely on monitoring the Earth’s environment.^2^ Mapping of the biophysical parameters of the land surface and the anthropogenic uses of land—or land cover and land use—has been undertaken for several decades now, made possible primarily through the availability of satellite imagery at increasingly higher spatial resolutions.^3^ The opening of the Landsat archive in 2010,^4^ freely available high-resolution Sentinel imagery since 2015 and Planet data for the tropics since 2020,^5^^,^^6^ and public access to very high-resolution satellite imagery through applications such as Google Earth and Microsoft’s Bing Maps,^7^ have all contributed to a proliferation of new high-resolution (10–30 m) products on land cover and land use, some of which are even dynamically updated as new satellite images become available.^8^

Artificial intelligence (AI) in the form of machine learning has been a part of the toolkit of remote sensors for decades.^9^ AI techniques are composed of a series of methods, tools, and algorithms that have been developed to loosely emulate some aspect of human intelligence or that perform tasks that require human-like intelligence. Machine learning is a specific branch of AI that focuses on methods that learn from data or past experiences to be able to, for example, interpret these data or to make predictions.^10^ This includes pattern recognition methods such as classification and clustering, which can be undertaken using different approaches such as neural networks, decision trees, support vector machines, etc., as well as conventional statistical models such as regression. Deep learning approaches, which are essentially further developments of neural networks,^11^ are now used in many different applications from computer vision (e.g., for image and facial recognition, and autonomous driving) to natural language processing (NLP) of large bodies of text. These methods can be trained using supervised, unsupervised, or reinforcement learning, but the task typically requires massive amounts of training data to ensure model robustness and high performance.

Various satellite sensors record multiple images in different parts of the electromagnetic spectrum and at different times, which, together with the spatial neighborhood context, offer a favorable higher-dimensional feature space for the application of AI machine learning approaches such as pattern recognition and classification. Supervised classification algorithms in remote sensing use training data derived traditionally from field-based data collection as well as expert visual interpretation of aerial and satellite imagery to produce different products for monitoring. However, the lack of training data remains one of the key challenges for improving Earth surface mapping.^12^ One alternative source of training data for remote sensing has been from citizen science, where citizens have used their cognitive skills in object recognition and visual interpretation to produce training datasets that are much larger than those traditionally produced by space agencies. For example, at the International Institute for Applied Systems Analysis (IIASA), we have developed a series of tools for crowdsourcing the visual interpretation of imagery and georeferenced photographs as well as the collection of *in situ* land cover and land use data. The Geo-Wiki^13^ and Picture Pile^14^ applications have been used to crowdsource large training and validation datasets on different types of land cover and land use but also the collection of other information such as the size of agricultural fields globally and the drivers of deforestation in the tropics.^15^ We have also developed the FotoQuest Go mobile app, which has directed users to specific locations on the ground to photograph and document land cover and land use.^16^ Other examples of crowdsourcing land cover information include National Aeronautics and Space Administration (NASA’s) Global Learning and Observation to Benefit the Environment (GLOBE) program, where a recent study showed how the protocol of data collection in multiple directions improved the land cover classification performance^17^ while OpenStreetMap contributors use very high resolution satellite imagery for recognizing and annotating features as well as damage mapping for humanitarian causes.^18^^,^^19^ The validation of automated change detection algorithms and the use of data contributed by citizens for updating an authoritative land cover dataset have also been achieved using volunteers.^20^^,^^21^

Such alternative and large data sources are particularly relevant for novel data-hungry AI approaches such as deep learning. However, there are limits to what the crowd alone can achieve. For this reason, AI is already being used in a number of citizen science projects to undertake two main types of tasks: recognition and prediction.^22^ Under recognition, this includes classification, counting objects, object detection and assessing the consistency of contributions by citizens. For example, the developers of the Galaxy Zoo application,^23^ which has classified millions of galaxies with volunteers,^24^ have trained a machine learning model with the data generated by their contributors to undertake classification. In this way, citizens are helping the AI to learn. At the same time, they continue to provide much needed inputs in cases where human cognitive skills are superior to the machine.^25^ They also use machine learning to assess the consistency of the annotations made by their citizen scientists.^26^ Other examples include the Serengeti Wildebeest Count,^27^ which uses deep learning for counting the number of wildebeests in images, and iNaturalist,^28^ where machine learning models provide suggestions of plant and animal species through automatic detection of objects in the photographs,^29^ thereby aiding citizens in undertaking their data collection tasks. Under prediction, tasks undertaken by AI include data correction of air quality sensor observations from citizens,^30^ the design of molecules in the EteRNA project,^31^ and predicting water quality in the FreshWater Watch project.^32^

Yet it is the recent advances in generative AI, in the form of multi-modal large language models (MLLMs) such as ChatGPT^33^ or the many open-source models available^34^ that hold considerable untapped potential for citizen science projects that involve classification tasks. While readers might be familiar with LLMS, MLLMs are LLMS that can take different forms of inputs (e.g., text, images and video) and output multi-modal information (e.g., take an image and output a description). With Petabytes of data now being generated through Earth Observation (EO), citizen science combined with generative AI could be used to exponentially increase the amount of *in situ* data available and improve the accuracy and timeliness of land cover and land use mapping in the future. In particular, tasks related to natural disasters and emergency response could benefit from the potentially increased speed offered by AI applications. A recent review of foundation models, which include MLLMs, found that the application of MLLMs in the field of remote sensing is under-explored, with more attention applied to visual foundation models (VFMs) and visual language models (VLMs),^35^ both of which could also be used in citizen science projects.

Here we present a demonstration of the capability of MLLMs for visual interpretation of satellite imagery and how these models could be integrated into a citizen science application like Geo-Wiki, exploiting the synergies between MLLMs and citizens. We then look at advances in the area of remote sensing foundation models (RSFMs) for how more spatially aware AI tools could improve the way we monitor the Earth’s surface.

## Citizen science and visual interpretation

**Figure 1:**
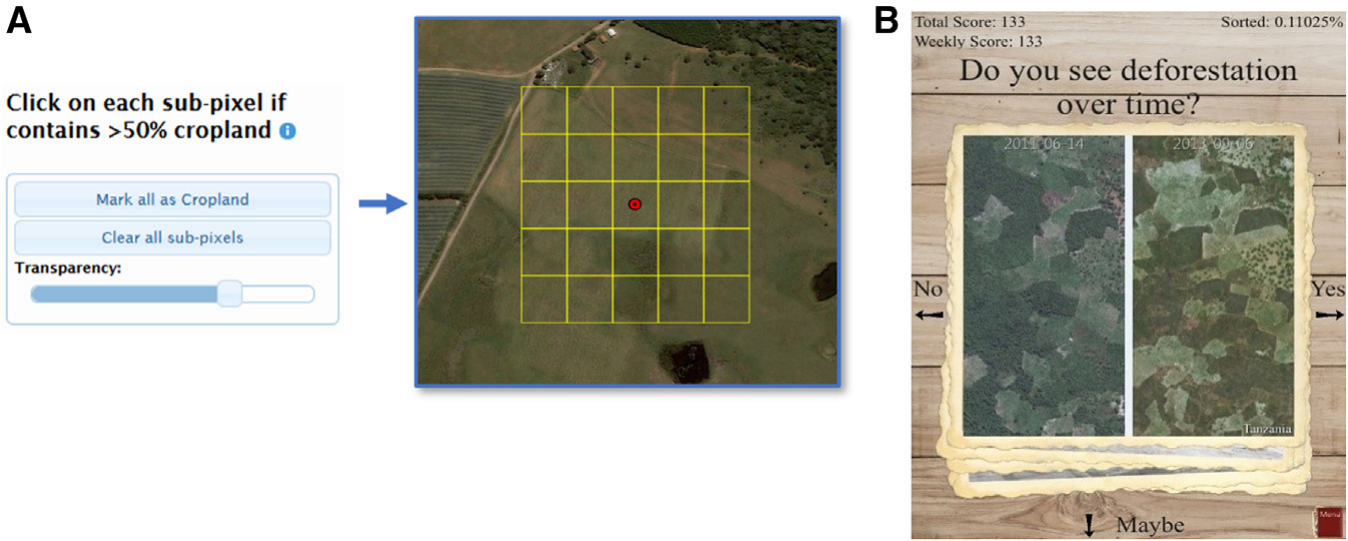
Examples of visual interpretation tasks undertaken by citizens Collection of data on (A) the presence of cropland using the Geo-Wiki desktop application interface and (B) deforestation using the the Picture Pile mobile application interface.

For the last decade, we have been involving citizen scientists in the visual interpretation of satellite imagery and the rapid labeling of georeferenced photographs to collect training data or validate existing land cover products.^14^^,^^15^ However, to date, we have not used AI for automated classification or to aid visual interpretation in any of our citizen science campaigns. To provide illustrative examples of the types of tasks that volunteers do as part of these campaigns, Figure 1A shows a screenshot from Geo-Wiki where volunteers examine satellite imagery and answer a series of questions that use their cognitive skills in recognizing key landscape features.^36^ In this particular example, citizens shade the sub-pixels if they see visual evidence of cropland covering more than 50% of each area, which resulted in a large global dataset on cropland.^37^ In Figure 1B, the Picture Pile mobile application interface is shown for the rapid classification of scenes from satellite imagery or geotagged photographs.^14^ In the example shown here, citizens examined two images from two different points in time to look for visual evidence of deforestation, swiping the image to the right if evidence was found. In total, the two applications have collected different types of *in situ* data including land cover, agricultural field sizes, drivers of deforestation, and human impact at several thousand locations on the Earth’s surface.^14^^,^^15^ However, this approach relies entirely on human interpretation, where some visual interpretation tasks are much easier than others and would, therefore, lend themselves well to AI.

## The potential of MLLMs for labeling and classification

**Figure 2:**
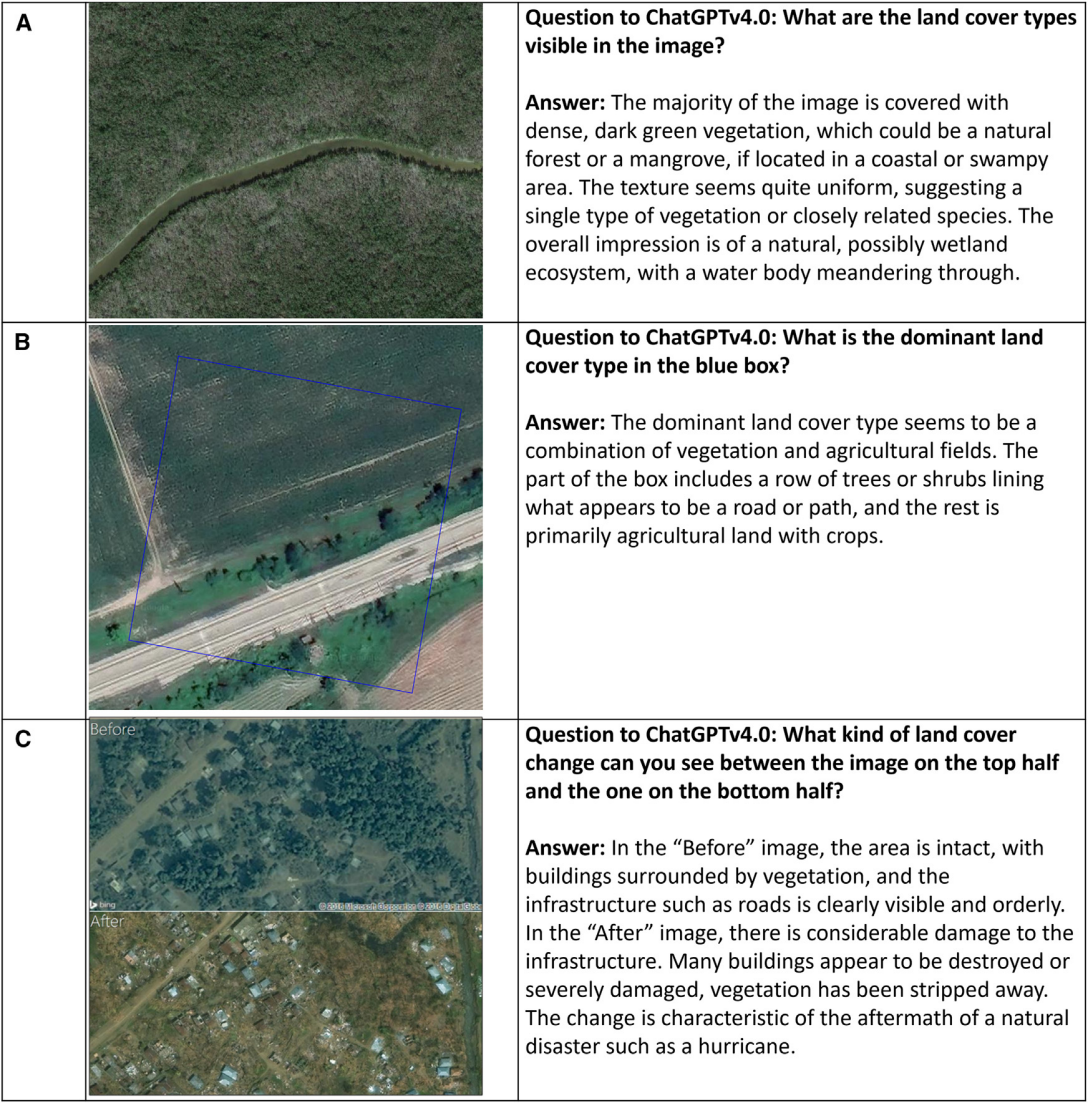
Examples of visual interpretation tasks undertaken by ChatGPT (A) A wetland/mangrove landscape in South America (B) an agricultural area in central Europe and (C) an area from Haiti before and after Hurricane Matthew that was part of a Picture Pile campaign. Source: Google Maps (A) and (B) and Microsoft Bing Maps (C).

MLLMs use deep learning and NLP in combination with massive datasets to infer relationships and generate new types of content.^38^ One notable feature of MLLMs is their ability to process different types of information, including images, and provide a description of what is contained in that image as an output, using other modules such as a pretrained vision transformer (ViT) like CLIP (contrastive language-image pretraining).^39^ This is analogous to what a visual interpreter would do when manually classifying scenes from satellite imagery for the collection of training and validation data. To demonstrate the potential of MLLMs for labeling and classification, we provided some visual interpretation tasks to ChatGPTv4.0 that are typical of the types of questions that we might ask volunteers in Geo-Wiki, shown in Figure 2 with ChatGPT’s responses. These are questions about the type of land cover that is visible in the image, the dominant land cover in an area that would be equivalent to a pixel (or grid cell) in a land cover map, and the change between two images from two different points in time.

In Figure 2A, ChatGPT has correctly identified a mangrove/wetland area, which is one of the land cover types that are often poorly identified in land cover maps and would benefit from considerably more training data. The task shown in Figure 2B is an example from an early Geo-Wiki campaign in which we asked volunteers to identify the dominant land cover type in a grid cell, indicated by the blue box. ChatGPT correctly picks out the dominant type as vegetation and agricultural fields but also identifies other relevant features in the image. Finally, Figure 2C shows two satellite images from Haiti before and after Hurricane Matthew hit the island in 2013. This was part of a Picture Pile campaign asking volunteers to rapidly identify building damage.^40^ Moreover, not only did ChatGPT identify the building damage, but it also inferred the potential cause as being some type of natural disaster such as a hurricane. This illustrates how MLLMs can be used to determine the type of change happening in images over time, where change detection is necessary for landscape monitoring. The production of land cover and land use time series that are spatially consistent over time is one area that needs further research^41^ so more *in situ* data on the spatial and temporal occurrence of land cover and land use change would be incredibly valuable for making advances in this area.

Using images from the Hurricane Matthew Picture Pile campaign (Figure 2C), we randomly selected 100 photographs where at least four out of six volunteers agreed that buildings showed signs of damage. We tested ChatGPT’s ability to detect evidence of building damage using the F1-score as a performance metric. The testing was repeated five times to evaluate stability, resulting in an average F1-score of 0.85 compared to the human-labeled damage data from the campaign. Further experimentation is still needed to assess the broader classification performance on a wider array of images. One potential strategy could involve prompt engineering. For example, a multi-step process might first identify whether there are buildings in the “after” image and then assess whether these buildings show signs of damage.

**Figure 3:**
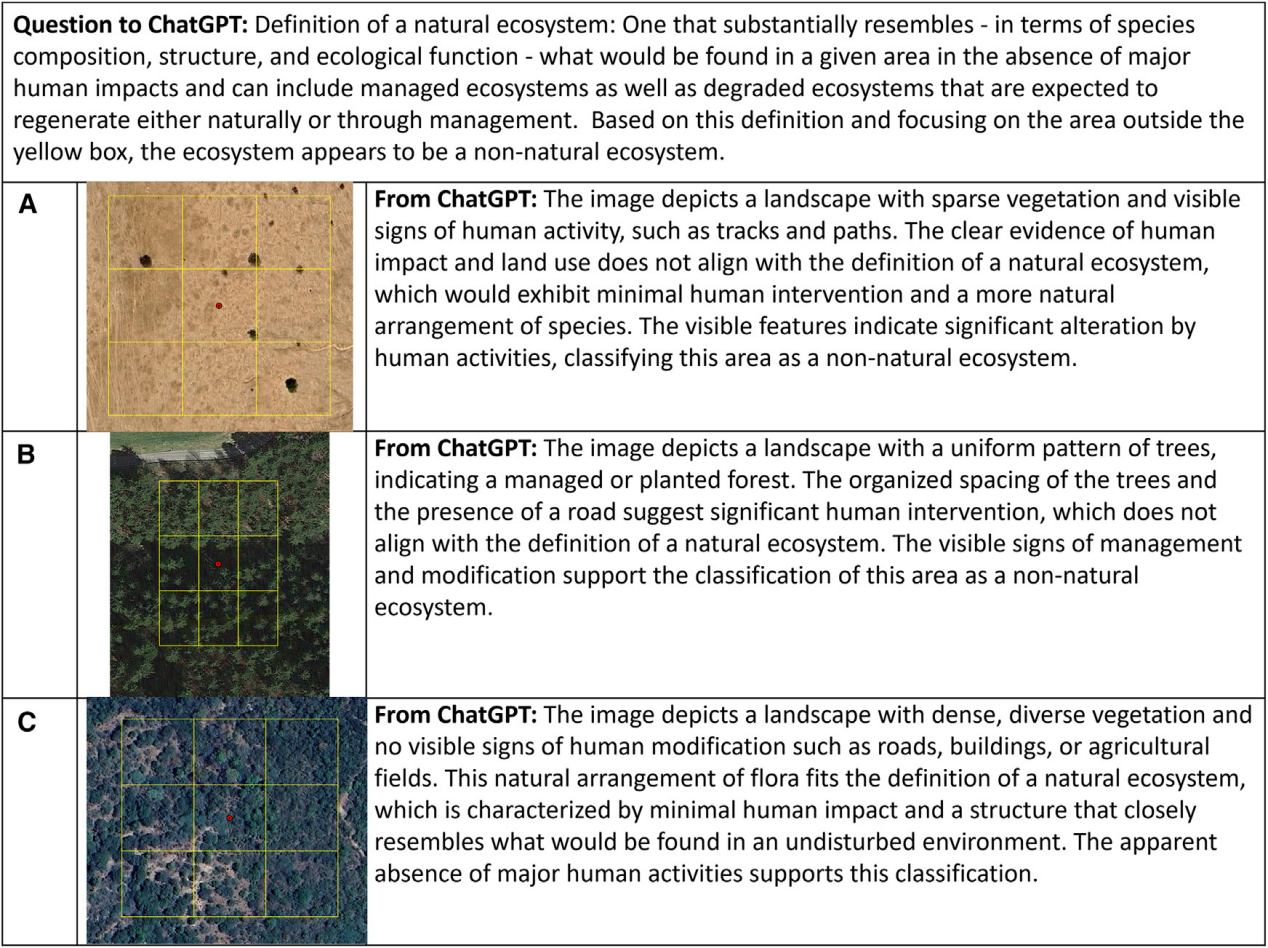
Examples of visual interpretation tasks undertaken by ChatGPT for identification of natural and non-natural ecosystems (A) and (B) shows examples where ChatGPT misclassified the images as non-natural for locations in Chad and Austria, respectively. In (C), the image from Colombia was classified as unsure by validators but classified as a natural ecosystem by ChatGPT. Source: Google Maps.

In another test with a more recent campaign on the identification of natural versus non-natural ecosystems for the validation of a natural ecosystem map, we gave 16 images to ChatGPT. The definition of natural landscapes was provided to ChatGPT as: one that substantially resembles—in terms of species composition, structure, and ecological function—what would be found in a given area in the absence of major human impacts and can include managed ecosystems as well as degraded ecosystems that are expected to regenerate either naturally or through management, based on the Accountability Framework initiative.^42^ The images were chosen to reflect a range of locations around the world with natural and non-natural landscapes, where the classifications were determined by a group of validators and checked via an expert in our group. Two images were also included that were classified as “Not sure.” The results are shown in Table 1. Most classifications were correct but there was confusion where ChatGPT classified the landscapes as non-natural when they were natural according to the validators; two examples are shown in Figures 3A and 3B. These explanations can be used to understand why there is confusion (e.g., is the track in Figure 3A made by humans or this natural?) or to improve the way in which the questions are asked (e.g., the road appears outside the yellow box in Figure 3B and should have been ignored). Hence this type of example can be useful in prompt engineering. Figure 3C shows one of the examples where the validators were unsure but ChatGPT chose a natural ecosystem. In this latter case the explanation provided by ChatGPT could be used to help classify images with uncertainty.

**Table 1:** Comparison of classifications from ground truth and ChatGPT for the natural ecosystems map validation campaign

Image	Location	Ground truth from validators	Classification from ChatGPT
1	China	Non-natural	Non-natural
2	Angola	Natural	Natural
3	Brazil	Natural	Natural
4	Colombia	Not sure	Natural
5	Nigeria	Non-natural	Non-natural
6	Chad	Natural	Non-natural
7	Sudan	Natural	Non-natural
8	Mali	Natural	Natural
9	India	Non-natural	Non-natural
10	Mexico	Natural	Natural
11	Egypt	Not sure	Natural
12	Iran	Natural	Natural
13	USA	Non-natural	Non-natural
14	Italy	Non-natural	Non-natural
15	Austria	Natural	Non-natural
16	Ukraine	Non-natural	Non-natural

Misclassifications are shaded in gray.

More recently, we demonstrated that ChatGPT could be used for mapping building attributes, which are land use features that are relevant to urban climate and energy balance modeling.^43^ Using a sample of 92 images of buildings from Manhattan (New York City), which were obtained from Mapillary, we asked ChatGPT to classify the buildings according to building function (residential, commercial, and mixed) and building age (Victorian, pre-WWII, post-WWI, postmodernist, and contemporary) as well as determining the building height. The overall accuracy for the prediction of building function and building age based on authoritative data from the city of New York and the Overture database were 79% and 56% respectively, while the average deviation in building height was around 3 feet. The main misclassifications were often due to obscured views so flagging these images and modifying the questions to ChatGPT could improve this performance in the future. However, further research is needed to determine how transferable such an approach would be to other cities, particularly those in the Global South.

## Integrating MLLMs in citizen science platforms for classification tasks

In the Geo-Wiki application, we primarily use very high-resolution satellite imagery, but this is only one of several sources of imagery that could be classified for land cover and land use. Aerial and drone imagery, as well as georeferenced *in situ* photographs are also now openly available. The LUCAS (Land Use/Cover Area frame Survey) database produced by Eurostat provides *in situ* photographs of land cover and land use at around 300K locations across European Union member states, taken every three years^44^ while Google Street View^45^ and Baidu Maps^46^ in China, for example, can provide global coverage of street level images. Various crowdsourced repositories of geotagged photographs also exist such as the Degree Confluence project,^47^^,^^48^ the Global Geo-Referenced Field Photo Library from the University of Oklahoma,^49^^,^^50^ Geograph^51^^,^^52^ and Mapillary,^53^ as well as social media sources like Flickr^54^ and Instagram.^55^ In addition to satellite imagery, we have used geotagged photographs from these types of sites for classification in Picture Pile.^14^

**Figure 4:**
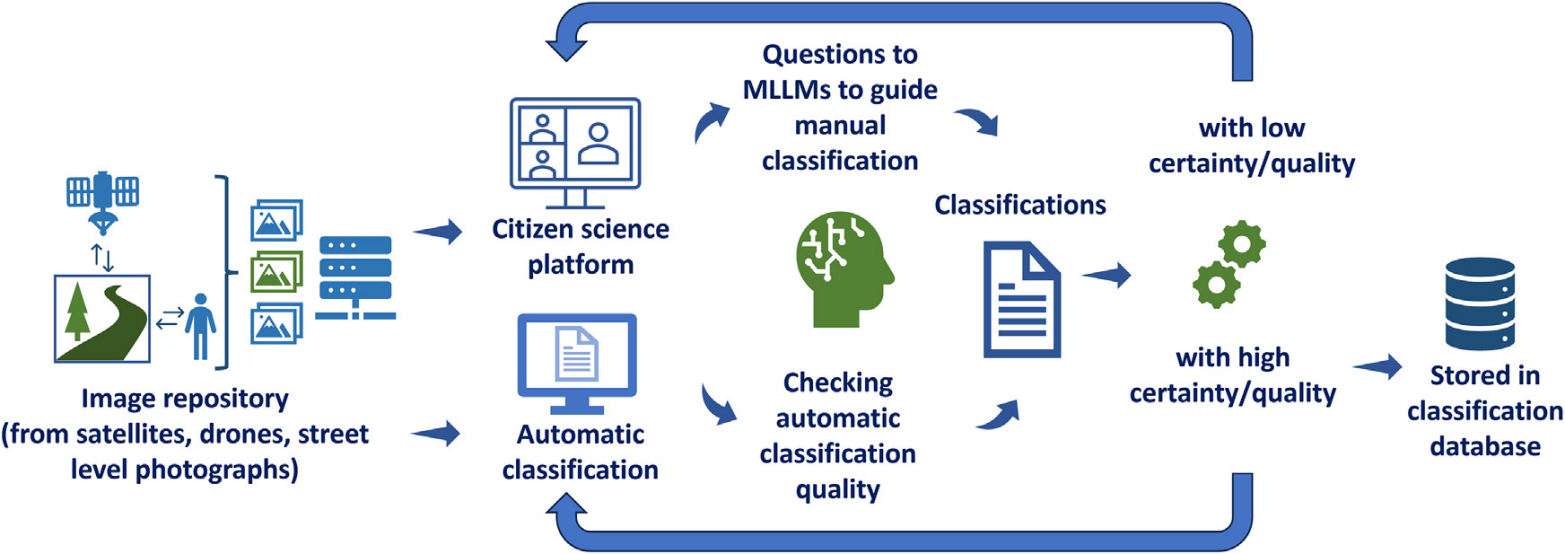
Integrating multi-modal large language models (MLLMs) in a citizen science visual interpretation workflow

Figure 4 shows how we might integrate MLLMs into a classification workflow that involves citizen science. After preparation of the image catalog, which could contain a range of different image types like that discussed previously, the images would be displayed in the citizen science platform. This would be integrated with an MLLM that would allow volunteers to ask relevant questions related to classification and labeling of land use and land cover to aid in their visual interpretation of the imagery. Guidance would be provided as to what types of questions could be asked but citizens could also share their experiences regarding how best to use this MLLM feature. In Geo-Wiki, citizens also indicate if they are unsure about their classification because the images were, for example, not clear or the features were not easily identifiable, which gives an indication of uncertainty.

At the same time, the MLLM (or a ViT) would be used to automatically classify the imagery, which could then be checked by citizens for quality. The advantage of using an MLLM like ChatGPT directly is the ease of use but the best solution would require more investigation including the use of other ViTs. The classifications from the citizens, which would include multiple instances of the same location, would be merged with the automatic classifications to filter out the high-quality data that would be written to the classification database. High quality would refer to locations where there was consensus between the citizens and/or the automatic classification and where no uncertainty was indicated. Ideally, the MLLM (or ViT) would also verbalize the level of uncertainty, which could then be used in the filtering process but research is still ongoing to find the best approaches to elicit reliable responses on confidence from MLLMs.^56^ Statistical methods would be employed to determine when enough classifications of the same location were made to reach the desired level of accuracy that would be specified by the application.^57^ Eventually more images would be automatically classified, with fewer and more difficult images provided to the crowd. This would mean that the time and efforts of citizens could be more optimally used to classify images that require their advanced human cognitive abilities while simultaneously contributing to the training and improvement of the MLLM (or ViT) and decrease the possibility of poor classifications or so-called hallucinations.^58^ Using such an approach, applications such as Geo-Wiki and Picture Pile could be enhanced, potentially resulting in the production of larger reference datasets for land cover/land use training and validation.

## Adding greater spatial awareness to AI

Since MLLMs are generally trained on existing human-generated content from the internet, they may have some spatial awareness. For example, they may have been trained on the existence of Tobler’s law, which states that features closer in space will be more related to one another than those further away,^59^ but they may not be able to apply it, or apply other geographical theories around how space is organized, when making inferences. To improve their spatial awareness, we could add other types of expert knowledge, for example, spatially explicit datasets such as species distribution models or bioclimatic ranges, or link to data from OpenStreetMap, which has already been demonstrated in the GeoLLM model.^60^ Other spatial and land cover/land use improvements could include adaptations to CLIP, such as RemoteCLIP^61^ that uses remote-sensing specific datasets, or GeoCLIP,^62^ that ingests geographic coordinates to produce a more geo-aware classification process.

**Figure 5:**
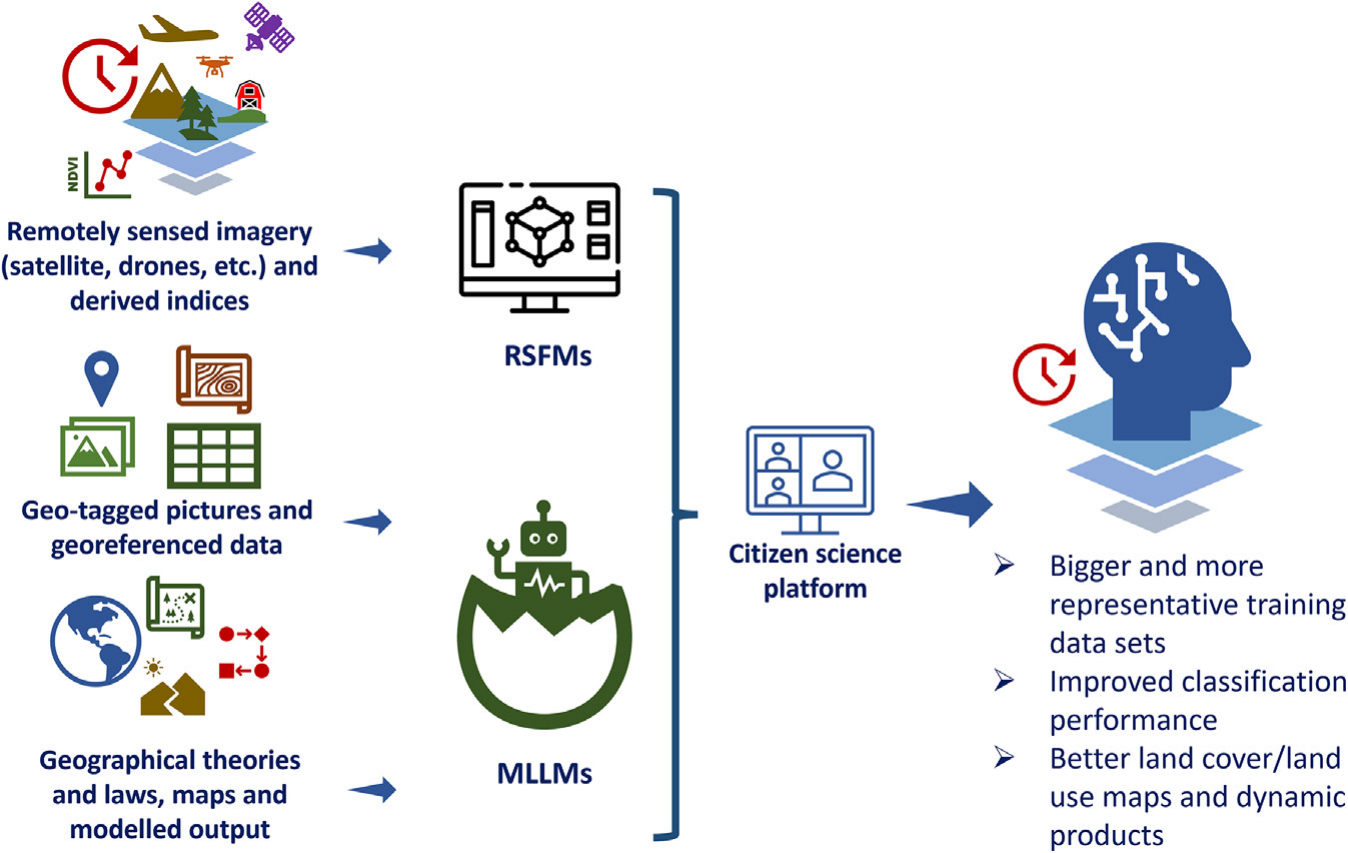
Integrating AI models (RSFMs and MLLMs) for improving land cover and land use mapping and reference data collection

Another important element of land cover and land use is monitoring the change over time, where remotely sensed time series data and derived indices are used. The incorporation of spectral information as well as data from multiple sensors is an active area of research in the field of RSFMs.^35^ Figure 5 shows a system whereby remote sensing data, ingested through RSFMs, and various types of spatial data, input to MLLMs, could be integrated with citizen science, broadening the workflow shown in Figure 4. Such an approach could result in more *in situ* data and ultimately to more accurate land cover and land use maps, including dynamic products.

## Opportunities, challenges, and other considerations

The use of generative AI in combination with citizen science and EO could result in a step change toward mapping the Earth’s surface more accurately and dynamically. The vision of an on-demand land cover mapping service as outlined by Szantoi et al.^12^ could even become a reality. Not only can land cover be mapped more efficiently but this opens up new possibilities for mapping land use and land use management. While these features are much more difficult to see from space, they can be captured, for example, in photographs. Creating good quality *in situ* datasets on land use and land use management with the help of generative AI may improve remotely sensed land use classification, particularly with the availability of imagery from the new generation of hyperspectral satellites.^63^

However, there are clearly challenges with such an approach. Realizing such a spatial-temporal AI trained with big spatial datasets as proposed here (Figure 5) would not be possible without a collaborative partnership between multiple institutions at an international level as well as considerable funding. Moreover, extensive testing of the capabilities of MLLMs and VLMs for visual interpretation would need to be undertaken to understand the limitations of the technology (e.g., what is the smallest mapping unit that would allow for accurate and reliable land use and land cover classification?), as well as how the tasks should be framed to produce high-quality results. Moreover, using commercial off-the-shelf MLLMs such as ChatGPT as demonstrated here could be very expensive depending on how many images are classified and what outputs are provided. A typical Geo-Wiki campaign classifies 100K–300K images; if this approach were to be extended to dynamic monitoring on a continuous basis, this could turn into millions of images on an annual basis. Hence, there is a need to investigate other open-source solutions.

Aside from the opportunities and specific research challenges highlighted previously, there are broader ethical and environmental implications of using generative AI more generally that should be considered. It is recognized that combining AI with citizen science may demotivate some volunteers so the distribution of tasks between AI and citizens requires careful design.^22^ In the citizen science and crowdsourcing that we implement, we pay particular attention to volunteer motivations (e.g., helping scientific research), and we provide incentives to participate (e.g., prizes and/or proper acknowledgment of contributions).^15^ However, there is a substantial, distributed labor force of data workers that provide labeling, feedback and moderation services that drive the development of AI,^64^ and there are exploitative practices and mental health problems that arise from this process.^65^^,^^66^ There are also environmental costs associated with training MLLMs^67^^,^^68^ that should be addressed given the urgent need to reduce greenhouse gas emissions and water usage. Hence, there are trade-offs in developing these types of models that should be properly assessed using tools that allow for more transparent accounting of the environmental footprints.^69^^,^^70^^,^^71^ But at the same time, we should continue to investigate the capabilities of MLLMs for improving global land mapping and monitoring in a way that is as sustainable and ethical as possible.

## Acknowledgments

This study has received funding from the European Union’s Horizon Europe Research and Innovation programme under grant agreement no. 101082130 (EVOLAND project) and no. 101059548 (OEMC project).

## Declaration of interests

The authors declare no competing interests.
